# A Web-Based Psychosocial Intervention for Family Caregivers of Older People: Results from a Mixed-Methods Study in Three European Countries

**DOI:** 10.2196/resprot.5847

**Published:** 2016-10-06

**Authors:** Francesco Barbabella, Arianna Poli, Frida Andréasson, Benjamin Salzmann, Roberta Papa, Elizabeth Hanson, Areti Efthymiou, Hanneli Döhner, Cristina Lancioni, Patrizia Civerchia, Giovanni Lamura

**Affiliations:** ^1^ Centre for Socio-Economic Research on Ageing National Institute of Health and Science on Ageing (INRCA) Ancona Italy; ^2^ Department of Health and Caring Sciences Linnaeus University Kalmar Sweden; ^3^ National Institute for the Study of Ageing and Later Life Linköping University Norrköping Sweden; ^4^ Swedish Family Care Competence Centre (NKA) Kalmar Sweden; ^5^ wir pflegen e.V. Berlin Germany; ^6^ Eurocarers Brussels Belgium; ^7^ Cyprus University of Technology Limassol Cyprus; ^8^ Neurology Unit National Institute of Health and Science on Ageing (INRCA) Ancona Italy

**Keywords:** caregivers, frail elderly, Internet, social support, social networking, health education

## Abstract

**Background:**

Informal caregiving is the main source of care for older people in Europe. An enormous amount of responsibility and care activity is on the shoulders of family caregivers, who might experience problems in their psychological well-being and in reconciling caregiving and their personal sphere. In order to alleviate such burden, there is increasing interest and growing research in Europe on Web-based support addressing family caregivers and their needs. However, the level of development and penetration of innovative Web-based services for caregivers is still quite low and the access to traditional face-to-face services can be problematic for logistic, availability, and quality reasons.

**Objective:**

As part of the European project INNOVAGE, a pilot study was conducted for developing and testing a Web-based psychosocial intervention aimed at empowering family caregivers of older people in Italy, Sweden, and Germany. The program offered information resources and interactive services to enable both professional and peer support.

**Methods:**

A mixed-methods, sequential explanatory design was adopted. Caregivers’ psychological well-being, perceived negative and positive aspects of caregiving, and social support received were assessed before and after the 3-month intervention. Poststudy, a subsample of users participated in focus groups to assist in the interpretation of the quantitative results.

**Results:**

A total of 94 out of 118 family caregivers (79.7%) from the three countries used the Web platform at least once. The information resources were used to different extents in each country, with Italian users having the lowest median number of visits (5, interquartile range [IQR] 2-8), whereas German users had the highest number (17, IQR 7-66) (*P*<.001). The interactive services most frequently accessed (more than 12 times) in all countries were the social network (29/73, 40%) and private messages (27/73, 37%). The pretest-posttest analysis revealed some changes, particularly the slight worsening of perceived positive values of caregiving (Carers of Older People in Europe [COPE] positive value subscale: *P*=.02) and social support received (COPE quality-of-support subscale: *P*=.02; Multidimensional Scale of Perceived Social Support subscale: *P*=.04), in all cases with small effect size (*r* range -.15 to -.18). Focus groups were conducted with 20 family caregivers and the content analysis of discussions identified five main themes: online social support, role awareness, caregiving activities, psychological well-being, and technical concerns. The analysis suggested the intervention was useful and appropriate, also stimulating a better self-efficacy and reappraisal of the caregivers’ role.

**Conclusions:**

The intervention seemed to contribute to the improvement of family caregivers’ awareness, efficacy, and empowerment, which in turn may lead to a better self-recognition of their own needs and improved efforts for developing and accessing coping resources. A major implication of the study was the finalization and implementation of the InformCare Web platform in 27 European countries, now publicly accessible (www.eurocarers.org/informcare).

## Introduction

A significant portion of adult individuals worldwide are experiencing increasing responsibilities of, and effects from being involved in, informal care for relatives with long-term care needs. For instance, it is estimated that the number of family caregivers over 18 years of age who care for older people and disabled adults are around 58 million in the European Union (EU) (15% of the adult population) [1] and 34 million (14% of the adult population) in the United States [2]. In the European context, the number of family caregivers is twice the entire health care workforce, with the economic value of informal care covering between 50% and 90% of overall costs for long-term care in EU member states [3]. The impact of caregiving on individuals’ lives is often remarkable and associated with different health and social issues. The prevalence of mental health problems among caregivers seems to be 20% higher than among noncaregivers [4], especially in terms of anxiety, depression, and distress attributed to their caring situation. Other risks for caregivers concern the possibility of encountering financial problems and difficulties in reconciling care with family activities and social life [4-6].

Recently, research in the European Union has increasingly concentrated on the development and testing of innovative solutions for providing support services to family caregivers of older people, especially in terms of Web-based programs with psychoeducational and psychotherapeutic purposes [7,8], or with multicomponent approaches including both professional and peer online support [9-11]. Although reviews in this field recommend more in-depth research for clarifying the effectiveness of Web-based interventions, preliminary evidence at the international level suggests that these should be multicomponent and tailored to caregivers’ actual needs and preferences, in order to impact effectively on caregivers’ psychological well-being, self-efficacy, and social inclusion [12-17]. This can be achieved by integrating the availability of information and educational modules with both professional and peer support, for instance, via interactive tools like discussion forums, chat rooms, and group videoconferencing [10,11,18-20].

So far, however, the level of development and coverage of Web-based programs for family caregivers has been rather low and fragmented in the European Union, with a number of small initiatives having limited scope and being sustained by poor funds and resources [21,22]. This fits into the broader picture of a general lack of formal support services dedicated to family caregivers [5,6]. This is further exacerbated in some EU countries—especially in Southern and Eastern Europe—by low policy, social, and cultural recognition of family caregivers’ roles, including a lack of legal rights, benefits, and support actions from public institutions and society [3].

As part of the wider INNOVAGE project, cofunded by the European Union, we addressed this systematic lack of online supports for caregivers by promoting a new social innovation at the European level (ie, an innovative solution to meet health and social needs of caregivers, to sustain their empowerment, and to improve their well-being) [23]. This social innovation was constituted by the new InformCare Web platform, which was intended to act as a first point of access to a variety of information, education, and social support opportunities at the country level for family caregivers, as well as an opportunity for formal services and nonprofit organizations in the field. Our research had the ultimate goal to implement the InformCare Web platform in 27 European countries in their official languages in order to allow caregivers from any involved nation to benefit from a set of minimum information and support. Research, development, and implementation activities were coordinated by the Italian National Institute of Health and Science on Ageing (INRCA) and the European nonprofit organization Eurocarers, with the support of the Swedish Family Care Competence Centre (NKA) and a wide network of national nonprofit organizations in the European Union.

Thus, this article reports the results from the pilot-testing in three European countries of a multicomponent, Web-based intervention delivered through the InformCare Web platform. The work is based on the assumption that caregiving activities can lead the caregiver to experience both negative feelings, such as subjective burden, stress, and depression [24,25], and positive ones, for instance, gain, reward, and satisfaction [26,27]. The goal of this pilot study was to verify the impact of the Web-based psychosocial intervention on caregivers, primarily in terms of benefits for psychological well-being, self-efficacy, and self-perception of both negative and positive aspects of caregiving, and secondarily as a potential driver of personal development and access to coping resources.

## Methods

### Design

The multicenter pilot study was conducted in Italy, Sweden, and Germany, and employed a mixed-methods, sequential explanatory design. Structured questionnaires with quantitative measures of the main outcomes were administered to enrolled family caregivers both at baseline and at 3-months postintervention; the study took place from April to July 2014. Postintervention, results from the structured questionnaires were used to organize a focus group in each country, at which a subgroup of users participated. The aim was to gain a more in-depth understanding of caregivers’ experiences, support the final analysis, and better interpret the results. The design and methods of the study were evaluated by competent local ethics committees in each country.

### Development of the Web Platform

The design and development of the Web platform was based on a review of the main needs and preferences expressed by family caregivers [5,28], as well as the areas of online health information and support [15,29-31]. A consultation process was also carried out via online surveys administered to 58 family caregivers, external experts, and stakeholders from different European countries, reached by means of national and international networks of partner organizations, in order to identify Web tools to include for addressing caregivers’ needs.

Individual user tests with 10 family caregivers were conducted on a first prototype of the Web platform in order to gain preliminary insights on its usability. Based on the feedback received, the platform was further refined for the pilot intervention.

### Intervention Conditions

**Figure 1:**
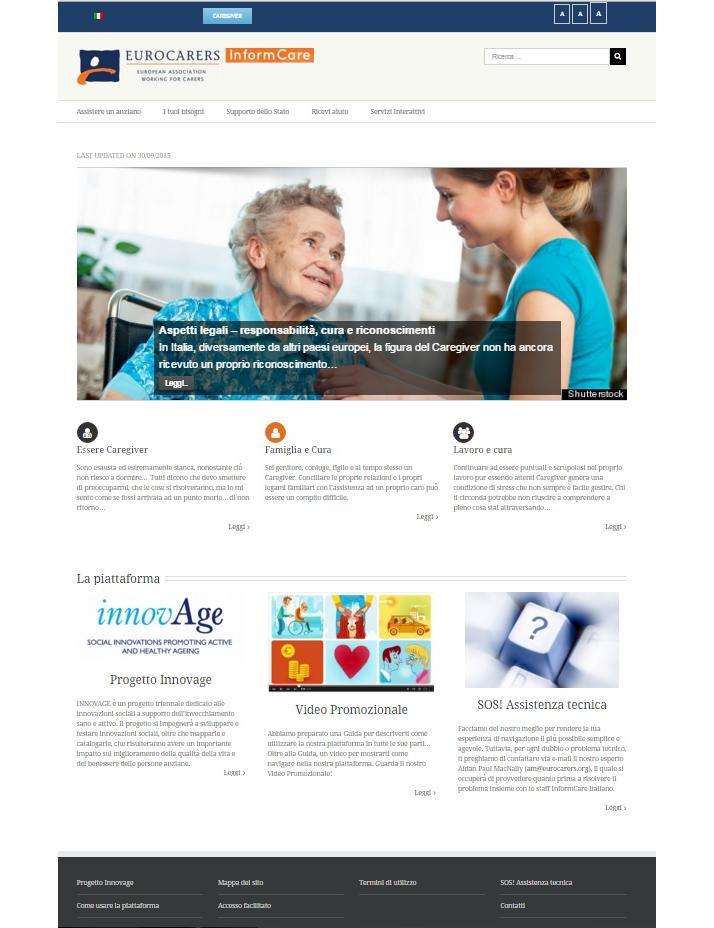
Screenshot of the home page of the InformCare Web platform for family caregivers of older people (example of Italian version).

In all three countries, the Web platform included both information resources and interactive services areas, developed in their national official languages. Access was restricted by means of an individual username and password given to each caregiver at the beginning of the study. An overview of the main characteristics of the information and services and a screenshot of the home page are provided in Table 1 and Figure 1, respectively.

Within the information resources area, four main sections were developed in order to improve knowledge and self-awareness, mainly concerning the caregiver’s role, coping strategies, and support available. Contents regarding the national range of services, benefits, and contacts available were appositely written by project staff and double-checked by external experts. Contents concerning general information on diseases, coping, and reconciliation strategies were provided by selected reliable websites in English managed by nonprofit organizations with a long-standing expertise in this field. Translation into national languages was carried out by national project staff and double-checked by senior project staff.

The interactive services area enabled communication among caregivers, as well as between caregivers and professional staff. The area included a set of Web tools: a dedicated social network, a forum, a private message feature, a chat feature, and a videochat feature. Interactive services were aimed specifically at improving caregivers’ psychological well-being, self-efficacy, and self-perception of caregiving situation. These services were delivered by means of individual and group online support provided in terms of information, advice, counseling, and emotional and social support. In each country, an interactive services area was managed by a professional moderator—a psychologist in Italy, and social workers in Sweden and Germany—who acted as an online counselor.

Some additional structured services and tasks were also proposed in order to better customize service provision to the sociocultural peculiarities and digital skills of national samples (see Table 1). The choice of services and tasks took into account that the main profiles of caregivers recruited in the three countries differed in terms of age and relationship with the older person, as well as of education and employment status, confirming what was highlighted by a previous European study [5,32].

Guidelines for moderators, who were trained prior to the intervention, were developed based on the main recommendations available in the field [33-35]. This aimed to clarify how support and interactions with users should be performed by moderators, and to set limits and standards of such support.

### Sample

The recruitment process adopted a convenience sample approach. Brochures and promotional materials were distributed in order to reach caregivers through available institutional and informal channels. In Italy, all participants were recruited through the Alzheimer Evaluation Unit at INRCA in Ancona, thus including family caregivers of people with Alzheimer’s disease or other dementias. In Sweden, caregivers were enrolled by exploiting existing networks of the NKA, the Swedish Dementia Association, Carers Sweden, and Linnaeus University in Kalmar and Växjö. In Germany, the nonprofit caregiver organization wir pflegen e.V. and local social care services contributed to recruitment by approaching family caregivers through their networks and websites.

Selection criteria for including family caregivers in the study were the following: (1) providing informal caregiving in activities of daily living (ADLs) and/or instrumental activities of daily living (IADLs) for an older person aged 60 years or more; (2) having basic digital skills, allowing the use of an Internet browser on a computer and/or mobile device; (3) having ordinary access to a computer and/or mobile device with Internet connection.

**Table 1:** Characteristics of information resources and interactive services.

Resources and services		Characteristics
**Information resources**		
	Caring for the older person	Symptoms, diagnosis, and treatments of the 10 most common chronic conditions Long-term care services at country level Environmental security
	Your own needs	Coping strategies Reconciliation with family and work Physical exercise
	Support by the state	Legal issues (eg, responsibility, rights, and competency) Economic and social insurance benefits
	Get help	Contacts for crisis or emergency List of relevant nonprofit associations List of other Web-based support programs
**Interactive services**		
	Social network	Channel: asynchronous group communication Possible user’s tasks: to see and read other caregivers’ profiles and posts; to post, comment, and share information and multimedia on personal and others’ walls Moderator’s role: to post regularly, both useful information (from the Web and the information resources area) and emotional statements; to interact with users by commenting on their posts and periodically leaving messages on their walls
	Forum	Channel: asynchronous group communication Possible user’s tasks: to open discussion threads on personal doubts and requests; to comment on others’ open threads Moderator’s role: to stimulate interactions by opening new discussion threads and/or commenting appropriately on users’ threads and comments
	Private messages/emails	Channel: asynchronous interpersonal or group communication Possible user’s tasks: to ask for direct support from the moderator; to contact other users Moderator’s role: to monitor and support users by sending periodical or ad hoc messages
	Chat and videochat/ videocommunication tools	Channel: synchronous interpersonal or group communication Possible user’s tasks: to ask for direct support from the moderator; to contact other users Moderator’s role: to support users by participating in individual or group discussions
**Additional structured services (country specific)**			
	E-learning course and virtual desk (Italy)	E-learning: multimedia training units with a focus on caregiving activities and long-term care services Virtual desk: weekly availability of moderator and other psychologists via chat, videochat, and forum for providing individual support
	Writing tasks in forum (Sweden)	Biweekly writing exercises alternating time management and emotional writing, managed by the moderator
	Videoconferencing groups (Germany)	Two weekly videoconferencing groups with three family caregivers each, managed by the moderator

### Procedure

At the outset, each participant caregiver signed an informed consent form and received a guide for accessing and using the platform in both paper and electronic versions. Preliminary face-to-face or videoconferencing meetings were organized on an individual or group basis for presenting and showing the platform.

Participants were invited to use the Web platform whenever they needed to find information, ask advice, or get support. Stimulation strategies were planned according to social and cultural preferences (eg, short message service [SMS] text messages and emails).

Technical support was guaranteed by both the moderator—for immediate help and clarification—and the Web developer—for fixing technical problems. A set of earphones was given to each caregiver allowing for the use of videochat and videocommunication tools.

### Quantitative Measures and Statistical Analysis

The primary outcome measures were represented by the caregivers’ psychological well-being and self-perception of both negative and positive aspects of caregiving. A secondary outcome was the social support the caregiver perceived from significant others and from services. Variables were measured with a structured questionnaire administered to all participants, both prior to and after the 3-month intervention, through an online system or, if requested, by post or email.

Sociodemographic characteristics and health problems of family caregivers and of their cared-for older persons—with the caregiver answering as a proxy—were asked through ad hoc categorical or binary questions. Other details were also asked about the care setting and access to public services (eg, home care and care allowances) and private services (eg, privately employed care assistant). As for the older person, ADLs and IADLs were measured, respectively, by means of the Barthel Index [36] (range 0-20, where 20 refers to a person who is independent in all activities) and the Duke Older Americans’ Resources and Services (OARS) scale [37] (range 0-6, where 6 is the highest number of activities for which the person needs help). Health status of the caregiver was assessed through a single item—health status 1 variable (HS1): self-perceived general health—retrieved from the Minimum European Health Module [38], whose results were recoded in three categories: good, fair, and bad.

Among the outcome measures, the 5-item World Health Organization Well-being Index (WHO-5) [39] was used to assess the level of psychological well-being perceived by the caregiver. It uses a 6-point Likert scale (ranging from *at no time* to *all of the time*) to rate statements such as “I have felt cheerful and in good spirits” and “I have felt calm and relaxed,” referring to the last 2-week period. Its percentage score was calculated by multiplying the raw score by 4 (ranging from 0 to 25).

The Carers of Older People in Europe (COPE) Index (15 items) [40,41] was included to ask about the perceived impact of the caregiving situation. The COPE Index uses a 4-point Likert scale (ranging from *never* to *always*) and includes three subscales concerning the following: negative impact, with seven items such as “Do you find caregiving too demanding?” (score range 7-28); positive value, with four items such as “Do you find caregiving worthwhile?” (score range 4-16); and quality of support, with four items such as “Do you feel well supported by friends or neighbors?” (score range 4-16). Negatively worded items of the negative impact subscale were reverse coded. A high score in a subscale indicated the following: low level of subjective burden (negative impact subscale); high level of positive feelings (positive value); and high level of support received by community, including family, friends, and formal services (quality of support).

Furthermore, the Multidimensional Scale of Perceived Social Support (MSPSS) (12 items) [42] measured the level of perceived social support received by the family caregiver. The MSPSS uses a 7-point Likert scale (ranging from *very strongly disagree* to *very strongly agree*) and includes three subscales asking to rate family (four items; eg, “My family really tries to help me”), friends (four items; eg, “I have friends with whom I can share my joys and sorrows”), and social support (four items; eg, “I have a special person who is a real source of comfort to me”). Each subscale ranges from 4 to 48 points, where 48 is the best support received, and a total score was calculated by summing the scores from all subscales.

Data about access to the information resources area of the platform were tracked through Google Analytics software. The number of times participants used single features of each interactive service were self-reported, then summed and categorized for each service as follows: never (no use), low use (6-12 times), and medium-high use (more than 12 times).

The Kolmogorov-Smirnov test was used to check for normal distribution of quantitative data. Data were expressed as frequencies for categorical variables, and as median (interquartile range [IQR]) and mean (SD) for continuous ones. Bivariate analysis was performed between the country variable and both sociodemographic characteristics and usage of the platform using the chi-square or Kruskal-Wallis tests for categorical or continuous variables, respectively. Comparison of paired data—medians before and after the intervention—on the primary outcome variables was carried out by the nonparametric Wilcoxon signed-rank test for dependent samples; effect size (*r*) was calculated as the Z value divided by the square root of the number of observations. A value of *P*<.05 was accepted as statistically significant. SPSS for Windows, version 16.0 (SPSS Inc) was used for the creation of the database, data cleaning, and data analysis.

### Focus Groups and Qualitative Data Analysis

A focus group was organized in each country after the intervention for further evaluation, especially with regard to the aspects of self-efficacy and support received online. Standard operative guidelines and a common set of topics to be covered were adopted for all focus groups, including the following: (1) appropriateness and usefulness of the intervention to meet own needs, (2) personal evaluation of using online services, and (3) perceived changes and improvements connected to the use of the services. Moderators of focus groups were senior project staff, whereas other trained researchers participated as observers and note takers.

Participants constituted a subgroup of the overall sample of family caregivers who used the platform. All focus groups took approximately 90-120 minutes; discussions were audiotaped and transcribed, with the support of field notes. Transcriptions and field notes were used for a conventional content analysis [43], based on a constant comparative approach [44], which aimed at exploring similarities and differences across the three country samples. By making systematic comparisons across units of data—participants’ comments and answers, and observations—researchers subsequently identified themes of discussion and selected relevant quotes from the focus groups [44,45]. Credibility of qualitative research was assured mainly by the following: prolonged engagement (eg, project staff’s long-standing experience of research and practice on Web-based support for caregivers); persistent observation (eg, direct knowledge gained by moderators and researchers on Web platform usage by caregivers, including types and frequency of peer and professional interactions); and peer debriefing, including the continuous involvement of an external advisory board (15 international experts) and the validation of final study results in an expert evaluation meeting (6 international experts) [46].

The analysis of qualitative data integrated quantitative results in an explanatory sequential process [47].

## Results

### Descriptive Statistics

Overall, 123 family caregivers were initially recruited to the study—59 in Italy, 44 in Sweden, and 20 in Germany—completing both the informed consent form and baseline questionnaire. A total of 5 participants dropped out during the intervention—1 in Italy, 3 in Sweden, and 1 in Germany—due to the death of the older person, changed life circumstances, or lack of time. At the end of the intervention, 94 out of 118 caregivers (79.7%) had accessed the Web platform at least once—42 in Italy, 36 in Sweden, and 16 in Germany.

Table 2 describes the sociodemographic characteristics of both the participating caregivers and the older persons they cared for. The median age of the older persons in the total sample was 80 years (IQR 74-85), the group consisted of mostly women in both Italy and Germany (79% and 69%, respectively), and they had different levels of ADL and IADL dependency.

Family caregivers who used the Web platform at least once were mostly women (64/94, 68%) with a median age of 58 years (IQR 51-69). In Italy, participants were mostly children and children-in-law of the older person, with low confidence with the Internet and high participation rates in the labor market, providing medium-low intensity of informal care. German caregivers were mostly unemployed children or children-in-law with a medium-high education, whereas the Swedish subsample included mostly retired spouses with high education.

### Usage

Table 3 shows how access to the two areas of the Web platform varied across countries. In general, the majority of caregivers in all countries accessed the platform two or more times—74% in Italy, 83% in Sweden, and 94% in Germany.

Concerning the information resources area, German and Swedish users made more visits than Italian ones in absolute terms (*P*<.001). The median number of visits ranged between 5 (IQR 2-8) in Italy and 17 (IQR 7-66) in Germany, with relevant differences in the number of pages visited (*P*=.001) and overall time spent (*P*=.002). In terms of interactive services, social network and private messages were used by the majority of participants. In particular, Swedish caregivers tended to use the forum more often (58% overall), followed by the chat feature (62%), and the videochat feature or other videocommunication tools (31%). German users displayed similar behaviors, whereas Italian caregivers hardly used the videochat feature (only 3% accessed it) and reported lower levels of access to both the forum and the chat feature (20% and 26%, respectively).

**Table 2:** Characteristics of older persons, family caregivers, and care settings by country.

Participant and setting characteristics		Italy (n=42)	Sweden (n=36)	Germany (n=16)	Total (n=94)	*P*^a^ value
**Older person**						
	Gender (woman), n (%)	33 (79)	13 (36)	11 (69)	57 (61)	<.001
	Age (years), median (IQR^b^)	82 (76-87)	76 (72-82)	82 (69-87)	80 (74-85)	.03
	ADL^c^ index, median (IQR)	15 (11-18)	11 (4-17)	5 (1-8)	13 (5-18)	<.001
	IADL^d^ index, median (IQR)	4 (2-6)	4 (2-6)	6 (4-6)	4 (2-6)	.05
**Family caregiver**							
	Gender (woman), n (%)	28 (67)	26 (72)	10 (63)	64 (68)	.73
	Age (years), median (IQR)	53 (47-58)	68 (57-73)	56 (53-67)	58 (51-69)	<.001
**Relationship to the older person,** **n (%)**						<.001
	Spouse/partner	1 (2)	27 (75)	3 (19)	31 (34)	
	Child/child-in-law	31 (74)	7 (19)	10 (63)	48 (51)	
	Other	10 (24)	2 (6)	3 (19)	15 (16)	
	Children (yes), n (%)	31 (74)	30 (83)	9 (56)	70 (75)	.20
	Grandchildren (yes), n (%)	10 (24)	22 (61)	2 (13)	34 (36)	<.001
**Health status, n (%)**						.11
	Bad	1 (2)	4 (11)	2 (13)	7 (7)	
	Fair	13 (31)	17 (47)	8 (50)	38 (40)	
	Good	28 (67)	15 (42)	6 (38)	49 (52)	
**Education, n (%)**						<.001
	Low	9 (21)	3 (8)	2 (13)	14 (15)	
	Medium	25 (60)	7 (19)	5 (31)	37 (39)	
	High	8 (19)	26 (72)	9 (56)	43 (46)	
	Employment (yes), n (%)	25 (60)	15 (42)	4 (25)	44 (47)	.04
**Living status (with respect to cared-for person), n (%)**						.002
	Same household	9 (21)	24 (67)	8 (50)	41 (44)	
	Within walking distance	13 (31)	4 (11)	5 (31)	22 (23)	
	Beyond walking distance	20 (48)	8 (22)	3 (19)	31 (33)	
**Confidence with Internet, n (%)**					<.001
	None/low	10 (24)	1 (3)	1 (6)	12 (13)	
	Medium	24 (57)	8 (22)	8 (50)	40 (43)	
	High	8 (19)	27 (75)	7 (44)	42 (45)	
**Care setting**							
	Informal care provided per week (hours), median (IQR)	12 (6-24)	32 (6-70)	30 (9-144)	15 (6-40)	.02
	Duration of caregiving period (years), median (IQR)	3 (2-4)	3 (2-7)	7 (2-9)	4 (2-5)	.09
	Home care (yes), n (%)	2 (5)	14 (39)	6 (38)	22 (23)	<.001
	Cash allowances received by older people and/or family caregivers (yes), n (%)	22 (52)	4 (11)	12 (75)	38 (40)	<.001
	Privately employed care assistant (yes), n (%)	18 (43)	11 (31)	7 (44)	36 (38)	.38

^a^Results of chi-square or Kruskal-Wallis tests for categorical and continuous variables, respectively. Sum of percentages may not be 100% because of rounding.

^b^IQR: interquartile range.

^c^ADL: activities of daily living.

^d^IADL: instrumental activities of daily living.

**Table 3:** Usage of the online information resources and interactive services by country.

Usage of resources and services			Italy (n=42)	Sweden (n=36)	Germany (n=16)	Total (N=94)	*P*^a^ value
**Information resources area, median (IQR^b^** **)**							
		Number of visits	5 (2-8)	13 (3-41)	17 (7-66)	7 (2-20)	<.001
		Number of pages visited	123 (75-186)	267 (66-790)	423 (121-926)	157 (67-362)	.001
		Time spent (minutes)	102 (53-163)	177 (65-755)	432 (113-689)	139 (57-405)	.002
		Pages per visit	25 (18-40)	20 (13-34)	21 (11-25)	22 (14-35)	.07
		Time per visit (minutes)	24 (16-30)	19 (10-27)	19 (10-25)	21 (14-29)	.048
**Interactive services area (Italy n=35; Sweden n=26; Germany n=12; total n=73), n (%)**							
	**Social network**						.001
		Never	20 (57)	2 (8)	4 (33)	26 (35)	
		Low use	8 (23)	8 (31)	2 (17)	18 (25)	
		Medium-high use	7 (20)	16 (61)	6 (50)	29 (40)	
	**Private messages**						.007
		Never	16 (46)	4 (15)	6 (50)	26 (36)	
		Low use	12 (34)	8 (31)	0 (0)	20 (27)	
		Medium-high use	7 (20)	14 (54)	6 (50)	27 (37)	
	**Forum**						.006
		Never	28 (80)	11 (42)	5 (42)	44 (60)	
		Low use	6 (17)	7 (27)	5 (42)	18 (25)	
		Medium-high use	1 (3)	8 (31)	2 (16)	11 (15)	
	**Chat**						.02
		Never	26 (74)	10 (38)	7 (58)	43 (59)	
		Low use	8 (23)	8 (31)	3 (25)	19 (26)	
		Medium-high use	1 (3)	8 (31)	2 (17)	11 (15)	
	**Videochat/ videocommunication tools**						<.001
		Never	34 (97)	18 (69)	5 (42)	57 (78)	
		Low use	1 (3)	4 (15)	2 (16)	7 (10)	
		Medium-high use	0 (0)	4 (15)	5 (42)	9 (12)	
**Specific country services or tasks, n (%)**							
		E-learning course (Italy) (yes)		25 (60)	N/A^c^	N/A	N/A	
		Writing task in forum (Sweden) (yes)		N/A	8 (22)	N/A	N/A	
		Videoconferencing sessions (Germany) (yes)		N/A	N/A	6 (38)	N/A	

^a^Results of chi-square or Kruskal-Wallis tests for categorical and continuous variables, respectively. Sum of percentages may not be to 100% because of rounding.

^b^IQR: interquartile range.

^c^N/A: not applicable.

**Table 4:** Impact of the Web-based intervention on users.

Outcomes		Baseline measurement (T0)		Postintervention measurement (T1)		*P*^b^ value	Effect size, *r*^c^
		Mean (SD)	Median (IQR^a^)	Mean (SD)	Median (IQR)		
WHO-5^d^		44.5 (24.2)	40 (24-60)	43.4 (23.0)	40 (24-60)	.41	-.06
**COPE^e^** **Index**							
	Negative impact	20.9 (4.1)	21 (19-24)	20.4 (4.2)	21 (18-23)	.22	-.09
	Positive value	12.6 (2.2)	13 (11-14)	12.1 (2.1)	12 (11-14)	.02	-.18
	Quality of support	10.4 (2.8)	10 (8-12)	9.8 (2.7)	9 (8-12)	.02	-.18
**MSPSS^f^**							
	Family	21.0 (5.8)	22 (17-26)	20.1 (5.9)	20 (16-25)	.04	-.15
	Friends	17.8 (6.1)	18 (13-23)	17.5 (6.1)	18 (13-22)	.71	-.03
	Social support	21.9 (5.8)	23 (18-27)	21.1 (5.5)	21 (17-26)	.04	-.16
	Total score	60.7 (14.2)	63 (50-73)	58.7 (14.2)	60 (47-69)	.11	-.12

^a^IQR: interquartile range.

^b^Wilcoxon signed-rank test for dependent samples, calculated between median values before and after the intervention.

^c^Effect size, *r*, is calculated as the Z value divided by the square root of the number of observations.

^d^WHO-5: 5-item World Health Organization Well-being Index.

^e^COPE: Carers of Older People in Europe.

^f^MSPSS: Multidimensional Scale of Perceived Social Support.

### Outcomes

Primary and secondary outcomes were assessed before (T0) and after (T1) the 3-month Web-based intervention (see Table 4). At baseline, the median scores of negative impact and positive value COPE subscales were relatively high (13 out of 16 points, negative impact; 21 out of 28 points, positive value), indicating quite low levels of subjective burden and a high positive experience of caregiving. The level of perceived social support was moderate, as suggested by midrange values in the COPE quality-of-support subscale and MSPSS, whereas the level of psychological well-being was quite low (median 40 out of 100 in the WHO-5 Index).

Concerning the pretest-posttest scores, the analysis showed that participants changed their perception toward different aspects. There was a statistically significant decrease of values concerning the positive value of caregiving (-1; *P*=.02) and the quality of support received by significant others (-1; *P*=.02) (COPE Index subscales), as well as by family (-2; *P*=.04) and social support in general (-2; *P*=.04) (MSPSS subscales). A small effect size [48] was found for all significant variables (ranging from -.15 to -.18). The scores concerning the other scales related to the negative impact of caregiving, support by friends, and psychological well-being showed no changes in values.

### Content Analysis of Focus Groups

A total number of 20 caregivers attended the focus groups: 7 in Italy, 7 in Germany, and 6 in Sweden. All participants in the three countries generally had a positive and satisfying experience with the platform, although there were slight differences in the emphasis of certain aspects. Data analysis identified five main themes: online social support, role awareness, caregiving activities, psychological well-being, and technical concerns. Theme analysis and relevant quotes are provided below (users’ names are fictional).

#### 1. Online Social Support

A consensus across the three focus groups was reported about the positive effects on social inclusion and support derived from using the interactive services. The platform was perceived as a safe virtual environment, which addressed caregivers’ needs to communicate with others and share personal experiences, more than any other available, mainstream, open-access social network (ie, Facebook). The possibility to interact in a protected environment with other people experiencing similar issues—although users did not know each other at first—led to increased mutual learning and understanding, as well as the recognition of not being alone in this condition. Both group and individual support provided by professional counselors was considered optimal and brought clear benefits. In particular, Swedish caregivers openly described that social recognition and confirmation by peers was useful for raising their own self-esteem, mastery over life, and sense of competence.

On other platforms, when I write something about my situation I have to explain. On this platform I don’t need to explain why I feel like I do, the other caregivers understand and know we have difficult times now and then.

Nils, Swedish adult son

Even just knowing that these kinds of support services exist and trustworthy people are working behind them, it is really important and helpful for family caregivers.

Patrizia, Italian adult daughter

#### 2. Role Awareness

Caregivers expressed that they felt a change in their understanding of their caregiving situation, claiming especially of having been stimulated to reflect about and understand more their own condition and needs. In Italy and Germany, participants agreed that reading and sharing caregivers’ experiences was emotionally difficult, but helpful in order to understand and better appraise their roles. Furthermore, many caregivers expressed that they had a better understanding about the future development of the older person’s condition, and what they could expect to face in the months or years to come. In Sweden, older female spouses emphasized the valorization of their role as a direct effect of online interactions, one of them even reporting that she could now see her activity more as a proper “job” and better accept this role.

You got the impression that you are understood, able to talk openly and got to reflect on your own situation. This motivates you to take on new steps to improve your personal situation.

Stefanie, German adult daughter

I have felt that my experiences are worth something, that I am not only an old lady in her 70s who should just sit and be quiet.

Lisa, Swedish older female spouse

#### 3. Caregiving Activities

Most caregivers in Italy and Germany underlined that information available on the platform, as well as tips and advice from other users, were useful to improve caregiving activities and better approach the cared-for person. Talking retrospectively, many users said their lives could have changed if they had had access to the platform earlier, because it could have helped them to recognize certain symptoms and help provide the older person with more adequate care.

My caregiving situation has improved by the tips I got from the other caregivers.

Phillip, German older male spouse

If this Web platform existed when our mum looked upset without any clear reasons, we would have realized more easily what she needed and would have avoided her having to suffer so much.

Roberta, Italian adult daughter

#### 4. Psychological Well-being

In terms of subjective well-being, some users in Sweden (both children and spouses) said they felt less burdened after the intervention, especially because they could better accept both positive and negative feelings arising from the caregiving situation. The possibility to express and share them with others, without being judged but rather receiving social recognition and confirmation of their own efforts by peers, helped them to cope with the situation and to reduce their perceived stress.

I feel happier and calmer when I can share the positive and negative things that happen in my situation as a caregiver. I don't know if I would have coped with the situation [without the platform] actually.

Nils, Swedish adult son

I feel less stressed and that can be a result of other people’s posts that I allow myself to have negative feelings and thoughts.

Anna, Swedish older female spouse

#### 5. Technical Concerns

 Despite the majority of participants who judged the usability of the platform as sufficient or good, some of them did mention technical or usability issues as a reason for not having used some of the available interactive services more. In particular, Swedish users reported problems with using the mobile version and specific features of some services (eg, uploading pictures on the social network, and using the chat and videochat features), whereas in Italy some caregivers found it difficult to find and reach some internal pages or services. In Sweden, an alternative videocommunication system was used with the moderator in order to overcome technical issues arising with the videochat feature. Support guaranteed by the moderator was in any case highly appreciated by all users across the three countries.

## Discussion

Results from our pilot study showed a statistically significant change of the perception by caregivers of some aspects related to the caregiving context. At the end of the intervention, caregivers reported slightly lower levels of positive feelings and social support received, whereas subjective burden and psychological well-being did not change. On the other hand, qualitative findings from the focus groups pointed out the usefulness and appropriateness of support received by caregivers from information and communication with moderators and peers. The major benefit for users seemed to be their empowerment, by means of increased self-efficacy, role awareness, and social recognition.

An interpretation of these ambivalent results can be that the intervention actually stimulated a new appraisal of the caregiving situation, including coping resources and social support available in the community, with caregivers recognizing ultimately a lack of adequate (external and/or professional) support from family, significant others, and formal services. The online information and support received via the platform could have produced a reappraisal of their own situation, thus allowing participants to identify more clearly and/or for the first time multiple issues of caregiving previously unrecognized, and to understand hidden needs for support.

Our results seem to be in line with previous international research in this field. Studies delivering multicomponent programs comparable to our intervention, that included unstructured support by professionals and peers, did not show significant changes in psychological well-being and burden [49], especially over a short time frame [9,11]. Overall, only some structured psychoeducational and psychotherapeutic programs were found to have an impact on caregiver burden and psychological well-being outcomes [7,18,19,50-52], whereas other studies highlighted mainly mixed or inconclusive results [8,53-56].

Furthermore, there is a lack of empirical literature regarding the effect of Web-based programs on perceived positive aspects of caregiving [13], which limits the possibility of comparisons with our partly unexpected findings. However, another short-term study found that some caregivers receiving the Web-based program had higher levels of stress at the end of the intervention than at baseline [8], a result that has been similarly explained as the possible consequence of caregivers’ enhanced awareness of their challenging caring situation. However, the worsening of positive feelings toward caregiving was narrow (median decreased from 13 to 12 in the 4-16 subscale range) and did not imply serious consequences for caregivers, also given the high level of initial scores and the qualitative findings collected in this respect.

Available literature also suggests that guidance from a professional counselor or coach is an effective way to address specific needs of caregivers [13,57]. As well, peer support in online communities can lead to increased confidence and self-efficacy [13-16,30], sense of belonging, and social inclusion [11,20,31]. Qualitative findings from our pilot study seemed to confirm these positive effects in the three countries, with major benefits for addressing social isolation for Swedish older spouses, also in line with available research [58].

Despite the lack of evidence in terms of burden and psychological well-being, the piloted intervention seemed able to provide useful and adequate online support services for family caregivers of older people, even in a short-term time frame, which might lead to increased efforts to alleviate stress by accessing coping resources and social support in the community [20,24,59]. In this respect, however, the challenge of tailoring the Web platform and tools to users’ digital skills and preferences represents a crucial issue to be considered for guaranteeing their usability and friendliness. This is especially true for caregivers with little experience of using Web services [10,15], as shown by the problems experienced by the Italian subsample, mainly due to low digital skills.

This study has some limitations to be taken into account and results cannot be generalized without caution. First, the study was conceived as a pilot test of a new Web-based program, able to carry out only a short-term and limited assessment of the intervention. Second, although the adopted mixed-methods approach gave the opportunity to integrate quantitative and qualitative results, associations between variables and causal relations could only be inferred. Third, despite the fact that the study was designed to include the main variables of interest, due to project constraints we could not include a control arm and cannot therefore exclude the influence of external variables on the outcomes. Fourth, only a subgroup of caregivers could be enrolled in the focus groups; indeed, we cannot fully exclude the influence of a selection bias in the qualitative findings. In general, the recruitment process was based on a convenience sample approach and bias in the profiles of the recruited caregivers was possible. Difficulties in approaching family caregivers [5], especially in testing Web-based services [7,8], are well-known in the literature, and they might have led to a slight imbalance of country subsamples concerning numbers and characteristics of caregivers involved.

Despite these limitations, it should be underlined that only a few studies have been able to involve similar or higher numbers of family caregivers in Web-based intervention research [7,9,19,50], and almost none have had a multi-country perspective [9]. Furthermore, the refinement and implementation of the InformCare Web platform at the European level within the INNOVAGE project was a direct consequence of this study. Based on results and indications from the pilot intervention, the project team managed an adjustment of information resources and a revision of guidelines for implementing and moderating interactive services, an effort conducted together with a network of appointed stakeholders—nonprofit organizations and experts—in the EU countries. This constitutes a remarkable, concrete added value of this research, since the platform has been accessible since mid-2015 in 27 EU countries via the Eurocarers website [60]. It includes 32 national versions, with some countries having more than one official language, and more than 2500 Web pages in the information resources area, which are publicly available and tailored to country characteristics. According to the availability and resources of national nonprofit organizations appointed in each country, a selection of interactive services may have been activated for national caregivers as well. Therefore, this study represents a unique example of translational research, which aims to contribute to the overcoming of social and cultural barriers for family caregivers that still exist in many countries by exploiting the potential of Web-based support. Future work might be based on this pilot experience and the implementation of the InformCare Web platform for conducting more in-depth and robust studies, especially on how to provide effective and tailored support for family caregivers, as well as for enabling cross-country, comparability research with a common set of intervention tools and guidelines.

## Abbreviations

ADL: activities of daily living
COPE: Carers of Older People in Europe
EU: European Union
FP7: Seventh Framework Programme
HS1: health status 1 variable
IADL: instrumental activities of daily living
INRCA: Italian National Institute of Health and Science on Ageing
IQR: interquartile range
MSPSS: Multidimensional Scale of Perceived Social Support
N/A: not applicable
NISAL: National Institute for the Study of Ageing and Later Life
NKA: Swedish Family Care Competence Centre
OARS: Older Americans’ Resources and Services
SMS: short message service
T0: baseline measurement point
T1: postintervention measurement point
WHO-5: 5-item World Health Organization Well-being Index
